# Glucuronide metabolites of *trans*-ε-viniferin decrease triglycerides accumulation in an in vitro model of hepatic steatosis

**DOI:** 10.1007/s13105-024-01035-w

**Published:** 2024-07-31

**Authors:** Pauline Beaumont, Samuel Amintas, Stéphanie Krisa, Arnaud Courtois, Tristan Richard, Itziar Eseberri, Maria P. Portillo

**Affiliations:** 1 UMR 1366, Univ. Bordeaux, Bordeaux INP, INRAE, ISVV, 33140 Villenave d’Ornon, OENO France; 2 UMR 1366, Bordeaux Sciences Agro, Bordeaux INP, INRAE, ISVV, 33170 Gradignan, OENO France; 3 INSERM U1312, Bordeaux Institute of Oncology - BRIC, BioGo Team, Bordeaux, France; 4 Service de Biologie Des Tumeurs Et Tumorothèque, CHU de Bordeaux, Bordeaux, France; 5 Centre Antipoison Et de Toxicovigilance de Nouvelle Aquitaine, Bâtiment UNDR, CHU de Bordeaux, 33076 Bordeaux, France; 6 Nutrition and Obesity Group, Department of Pharmacy and Food Sciences, Faculty of Pharmacy, University of Basque Country (UPV/EHU) and Lucio Lascaray Research Centre, 01006 Vitoria-Gasteiz, Spain; 7 Bioaraba Health Research Institute, 01009 Vitoria-Gasteiz, Spain; 8 CIBEROBN Physiopathology of Obesity and Nutrition, Institute of Health Carlos III (ISCIII), 28029 Madrid, Spain

**Keywords:** *Trans-*ε-viniferin, Resveratrol dimer, Glucuronide metabolites, AML-12-hepatocytes, Liver steatosis

## Abstract

*Trans*-ε-viniferin, a resveratrol dimer found mainly in grapevine wood, has shown protective capacities against hepatic steatosis in vivo. Nevertheless, this compound is very poorly bioavailable. Thus, the aim of the present study is to determine the potential anti-steatotic properties of 1 and 10 µM of *trans*-ε-viniferin and its four glucuronide metabolites in AML-12 cells treated with palmitic acid as an in vitro model of hepatic steatosis. The effect of the molecules in cell viability and triglyceride accumulation, and the underlying mechanisms of action by Real-Time PCR and Western Blot were analysed, as well as the quantification of *trans*-ε-viniferin and the identified bioactive metabolite inside cells and their incubation media. Interestingly, we were able to determine the triglyceride-lowering property of one of the glucuronides (*trans*-ε-viniferin-2-glucuronide), which acts on de novo lipogenesis, fatty acid uptake and triglyceride assembly. The glucuronides of *trans*-ε-viniferin would therefore be partly responsible for the in vivo observed anti-steatotic properties of the parent compound.

## Introduction

Liver steatosis, also known as fatty liver, is defined as an excessive fat accumulation in the liver, in amounts higher than 5% of liver weight, not related to alcohol intake. This entity is the most benign manifestation of Metabolic dysfunction-associated fatty liver disease (MAFLD), a hepatic disease characterised by an excess of 5% triglyceride accumulation in the liver and which is related to a systemic metabolic dysregulation which also encompasses steatohepatitis (non-alcoholic steatohepatitis, NASH), cirrhosis and cancer [26, 29]. Liver steatosis is a very prevalent hepatic alteration, which affects about 25% of the general population in Western countries [1]. Taking into account that there is no standardised treatment for liver steatosis to date [10], the study of natural compounds with potential beneficial effects is of definite interest in the aim of using them as complementary tool to prevent and/or treat this disease.

**Fig. 1:**
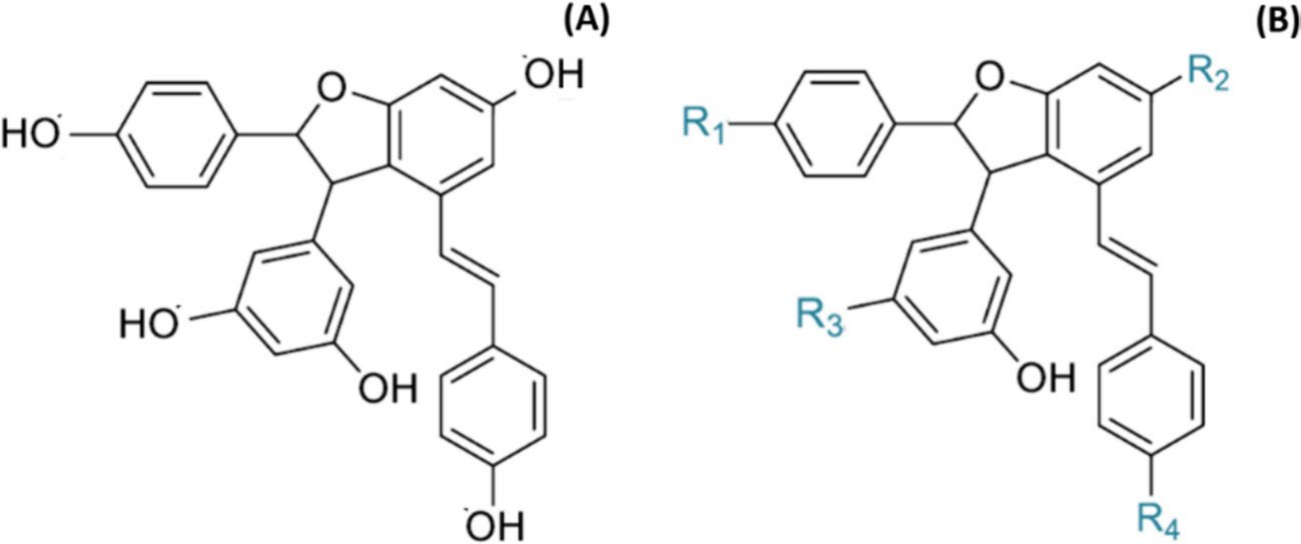
Structure of *trans*-ε-viniferin (**A**) and the four positions in which it can be glucuronided (R1-R4) (**B**)

*Trans*-ε-viniferin is a resveratrol dimer (Fig. 1A), which belongs to the group of stilbenes [27, 28]. This compound is found in many families of plants such as *Fabaceae, Cyperaceae, Iridaceae, Gnetaceae* and *Vitaceae* [18]. Concerning the latter family, it has been shown that it accumulates mainly in the woody parts of *Vitis vinifera* [19]. Thus, the use of these parts as a source of *trans*-ε-viniferin is a very interesting argument in favour of the principles of circular economy. For human consumption, *trans*-ε-viniferin is found in grapes and its derivative products, such as juices and red wine [6, 13]. Although its monomer, resveratrol, is the most studied stilbene for its numerous biological properties [17], *trans*-ε-viniferin also exhibits interesting bioactive functions as demonstrated in numerous studies [4]. More specifically, *trans*-ε-viniferin has revealed beneficial effects as anti-obesity, anti-diabetic, anti-steatotic or antihypertensive molecule [30, 32, 39, 46]. Concerning liver steatosis, Ohara et al. (2015) observed a reduction in hepatic triglyceride levels in mice fed with a high-fat diet supplemented with 0.2% of *trans-*ɛ-viniferin [39]. Furthermore, Liu et al. (2020) carried out a histological analysis in mice fed with a high-fat high-sucrose diet, and observed an amelioration in steatosis degree and inflammation in animals treated with *trans*-ɛ-viniferin at 30 or 60 mg/kg body weight/d, these effects being greater at the highest dose [30].

In spite of these beneficial effects, it is important to emphasise that *trans*-ε-viniferin is very poorly bioavailable [30]. In fact, Kim et al. estimated its bioavailability at 0.77% after an oral administration of 40 mg/kg body weight in mice [25]. This is explained, on the one hand, by a low intestinal absorption (measured in vitro with the Caco-2 cell model) [43] and, on the other hand, by a rapid phase II metabolism in the liver, which has been demonstrated in vitro on hepatic fraction of rats and humans [12] and in vivo [11]. As a matter of fact, in rats, a strong glucuronidation of *trans-*ε-viniferin takes place after intraperitoneal administration, especially into one isoform of the four possible conformations of *trans*-ε-glucuronides [11] (Fig. 1B). It has been demonstrated that encapsulation of *trans-*ε-viniferin in multilamellar liposomes leads to stronger glucuronidation and a more prolonged exposure time of the organism to glucuronide forms. In the previously cited study, the rat liver contained 60 times more glucuronides than the native form. This biotransformation is catalysed by the enzyme UDP-glucuronosyltransferase (UGT), which is present under several isoforms. Interestingly, the expression of the isoforms not only does it vary according to the species, but also to the sex and the tissue in which they are present [38, 40].

In this scenario, the question that arises is whether *trans-*ε-viniferin metabolites show biological activities. Thus, since we had previously observed that glucuronide metabolites of other stilbenes, such as resveratrol and pterostilbene, were involved in the decrease in triglyceride accumulation in a hepatocyte cell line (AML-12) [41, 42], we hypothesised that the glucuronide metabolites of *trans-*ε-viniferin could be totally or partially responsible for such an effect. Considering all this information, the present study aims to explore the potential contribution of the four glucuronides to the anti-steatotic effect of *trans-*ε-viniferin, in an in vitro model of hepatic steatosis, using palmitic acid (PA)-treated AML-12 cells.

## Material and methods

### Chemical and reagents

For cell culture, Dulbecco modified Eagles minimal essential medium (DMEM)/HAM’s F12 (F-12 Nutrient Medium) Glutamax, insulin, transferrin and selenium were obtained from Thermo Fisher (Waltham, MA, USA). Foetal bovine serum (FBS) was purchased from Corning (New York, NY, USA). Antibiotics (streptomycin and penicillin) and trypsin/EDTA were obtained from Lonza (Basel, Switzerland). The PA was purchased from Sigma-Aldrich (St Louis, MO, USA).

Concerning hemisynthesis and UPLC-DAD-MS analysis, acetobromo-α-Dglucuronic acid methyl-ester, pyridine and K_2_CO_3_ were purchased from Sigma-Aldrich (Saint Quentin Fallavier, France); and ultra-pure methanol, ethanol, acetonitrile, ethyl acetate and formic acid were purchased from Fisher Scientific. Milli-Q water used for these experiments was produced in the laboratory using Purelab Ultra System (Elga Lab Water, High Wycombe, UK).

### Stilbenes and metabolites production

*Trans*-ε-viniferin was extracted from the stems of *Vitis vinifera* as previously described [7], using solid/liquid and liquid/liquid extraction before separation and purification by counter-current chromatography and preparative high performance liquid chromatography, respectively. Purity was measured by UPLC-DAD-MS and ^1^H NMR, and was superior to 89% *trans*-ε-viniferin.

The four isoforms of *trans*-ε-viniferin-glucuronides were produced by hemisynthesis reaction previously developed with some modifications and using K_2_CO_3_ as base, as previously described [5, 12]. To purify a relatively large amount of the different compounds, the production was pre-purified by three consecutive liquid/liquid extractions with ethyl acetate/water (50:50, v:v) before drying the organic phase and injecting it in semi-preparative high performance liquid chromatography. Separation parameters used to purify the four compounds and non-transformed *trans-*ε-viniferin was the same as the one previously described [5].

### Cell culture and treatments

Mouse hepatocytes AML-12 were purchased from ATCC (ATCC®CRL-2254™, Manassas, VA, USA). They were allowed to grow in a medium made up of DMEM/HAM’s F12 Glutamax with 10% heat inactivated FBS, 5 µg/mL of insulin, 5 µg/mL of transferrin, 5 ng/mL of selenium, 40 ng/mL of dexamethasone and 1% of antibiotics (penicillin/streptomycin, 10,000 U/mL). The hepatocytes were maintained at 37 °C and 5% CO_2_ in 75 cm^2^ flasks. After the experimental period, cells were harvested with trypsin–EDTA for further analysis.

To induce triglyceride accumulation and therefore mimic hepatic steatosis, cells were exposed to 300 µM of PA for 18 h. The use of PA to induce triglyceride accumulation was based on the literature; several studies have demonstrated that both PA and oleic acid are effective for this purpose, and the mechanism for this effect is the stimulation of free fatty acid uptake in cells treated with both fatty acids [23, 36, 44, 45]. Moreover, the incubation conditions have been previously optimised to achieve significant accumulation of triglyceride without compromising cell viability [42]. For the treatment, hepatocytes were co-incubated for 18 h with PA, and the five molecules (*trans*-ε-viniferin and its four glucuronide isoforms, diluted in 95% of ethanol) at 1 µM and 10 µM. The experiments were run three times. To carry out the chromatographic studies of the molecules found in the culture media as well as inside the hepatocytes (*trans*-ɛ-viniferin and *trans*-ε-viniferin-2-glucuronide (V2G), the concentration of these molecules used for incubation was 10 or 25 µM, respectively, based on the detection limits of our devices.

### Viability tests and quantification of triglyceride content

For the viability test, Crystal Violet coloration was used to determine the living cells as it has been previously described [41]. Cell viability was determined in comparison with non-treated cells.

To quantify triglycerides, cells were washed with sterile PBS, harvested and sonicated [41], and the analysis was performed using a commercial kit (Spinreact, Girona, Spain) following the manufacturer´s instructions. Results were standardised by measuring the protein content of each cell using the Bradford method [8], and then expressed in mg of triglyceride per mg of protein.

### RNA extraction and Real-Time PCR

For RNA extraction, cells were washed with sterile PBS and harvested using Trizol (Invitrogen, Carlsbad, CA, USA). The RNA extraction, complementary DNA (cDNA) synthesis and Real-Time PCR reaction were performed as described in our previous publication [15]. Carnitine palmitoyl transferase 1a (Cpt1a), uncoupling protein 2 (Ucp2) and citrate synthase (Cs) expression was analysed using SYBR Green Master Mix (Applied Biosystems, Foster City, CA, USA) and the upstream and downstream primers (300 nM), and acetyl-CoA carboxylase (Acc), diacylglycerol acyltransferase 2 (Dgat2), Microsomal Triglyceride Transfer Protein (Mttp) and cluster of differentiation 36 (Cd36) were analysed using TaqMan® Gene Expression Assays following the manufacturer’s instructions. The mRNA levels of each gene were normalised to β-Actin values. The results were expressed as fold changes of threshold cycle (Ct) value relative to controls by the 2^–ΔΔCt^ method [31].

### Western blot analysis

30 µg of protein were separated by SDS-PAGE Electrophoresis in a 4–15% polyacrylamide gel (Bio-Rad, Hercules, CA, USA) and electroblotted onto PVDF membranes (Millipore, Bradford, MA, USA). Membranes were then incubated overnight with specific primary antibodies for Ser79 phosphorylated ACC (pACC, Cell Signalling Technology, Danvers, MA, USA), fatty acid synthase (FAS, Abcam, Cambridge, UK) and fatty acid transporter protein 2 (FATP2, Santa Cruz Biotech, CA, USA). Next, membranes were incubated with the secondary antibodies and detected using ChemiDoc™MP Imaging System (Bio-Rad, Hercules, CA, USA). The expression of each protein was normalised to α-tubulin (Santa Cruz Biotech, CA, USA) values.

### FAS activity

FAS activity was measured by a spectrophotometer at 340 nm of NADPH absorption, as previously described [33].

### UPLC-DAD analysis

For metabolic profile analysis, incubation media was removed 18 h after treatment initiation with molecules at 10 μM, and directly kept at -80 °C until analysis. Each sample was then slowly thawed. About 200 µL medium were rigorously vortexed and centrifuged (30 min × 10,000 g × 4 °C) before UPLC-DAD-MS analysis. In addition, incubation media for cells without treatment and media with molecules but without cells were analysed to evaluate potential co-elution and degradation, respectively. After washing AML-12 cells with ice-cold PBS, they were lysed with methanol. The cell extracts were evaporated with liquid N_2_, and the samples were then dissolved in methanol/H_2_O (50/50, v/v) and centrifuged 30 min × 10,000 g × 4 °C) before UPLC-DAD-MS analysis. UPLC separation was performed using a 1290 Infinity UPLC (Agilent Technologies, Courtaboeuf, France) with the same parameters previously described [42].

### RNAseq database analysis

Data analysis was carried out to compare the expressions of the different UGTs between the cell line of hepatocytes used for the experiments and a primary culture of hepatocyte of the same species (mouse). RNA-seq dataset was obtained from study GSE171184 [21] using GEO (https://www.ncbi.nlm.nih.gov/geo/). Raw genes RNA-seq counts for AML-12 cells and primary murine hepatocytes were normalised using the "median of ratios" normalisation method [2]. Log2 fold change p-values were calculated using Student’s *t* test and normalized RNA-seq counts.

### Statistical analysis

Results are presented as mean ± standard error of the mean (SEM). Statistical analysis was performed using SPSS 24.0 (SPSS Inc. Chicago, IL, USA). Analysed variables were normally distributed, according to Shapiro–Wilk´s test. Comparisons between each treated group of cells with either the control hepatocytes or the hepatocytes incubated with PA were performed using Student’s *t* test. Statistical significance was set up at the < 0.05 level.

## Results

### Effects of trans-ε-viniferin and the four trans-ε-viniferin glucuronides on cell viability and triglyceride content

**Fig. 2:**
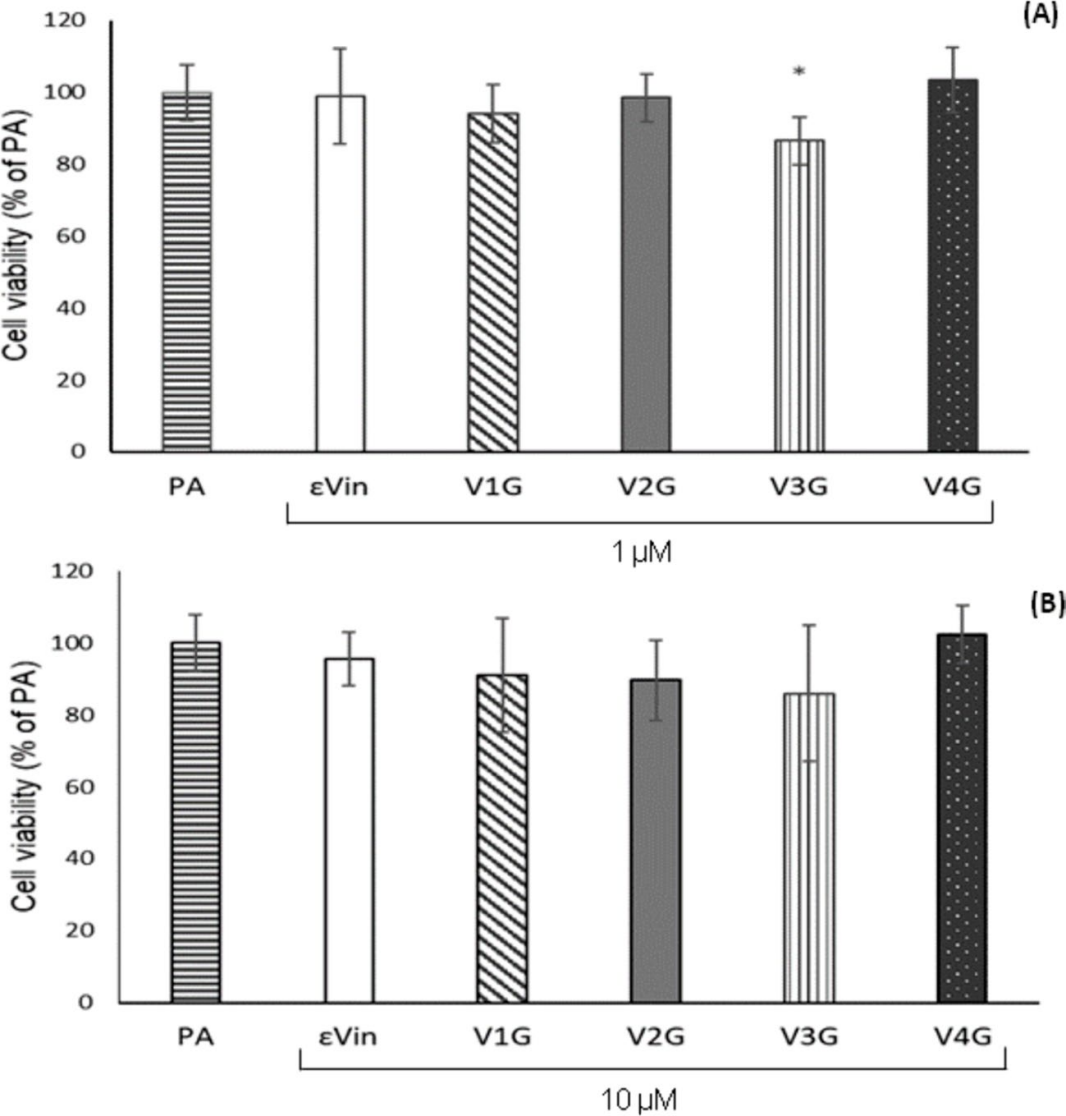
Cell viability in AML-12 hepatocytes exposed to palmitic acid (PA) with or without *trans*-ε-viniferin (ε-Vin), *trans*-ε-viniferin-1-glucuronide (V1G), *trans*-ε-viniferin-2-glucuronide (V2G), *trans*-ε-viniferin-3-glucuronide (V3G), or *trans*-ε-viniferin-4-glucuronide (V4G) at 1 µM (**A**) or 10 µM (**B**) for 18 h. Data are expressed as mean ± SEM. Each group was compared with PA-treated cells. *: *P* < 0.05

Crystal Violet assay was performed in cells exposed to *trans*-ε-viniferin and the four *trans*-ε-viniferin glucuronides (*trans*-ε-viniferin-1-glucuronide (V1G), V2G, *trans*-ε-viniferin-3-glucuronide (V3G), *trans*-ε-viniferin-4-glucuronide (V4G)), each compound at 1 or 10 µM in combination with PA for 18 h. The cell viability results are shown in Fig. 2. At 1 µM, only *tran*s-ε-viniferin-3-glucuronide (V3G) showed a slight cytotoxic effect.

**Fig. 3:**
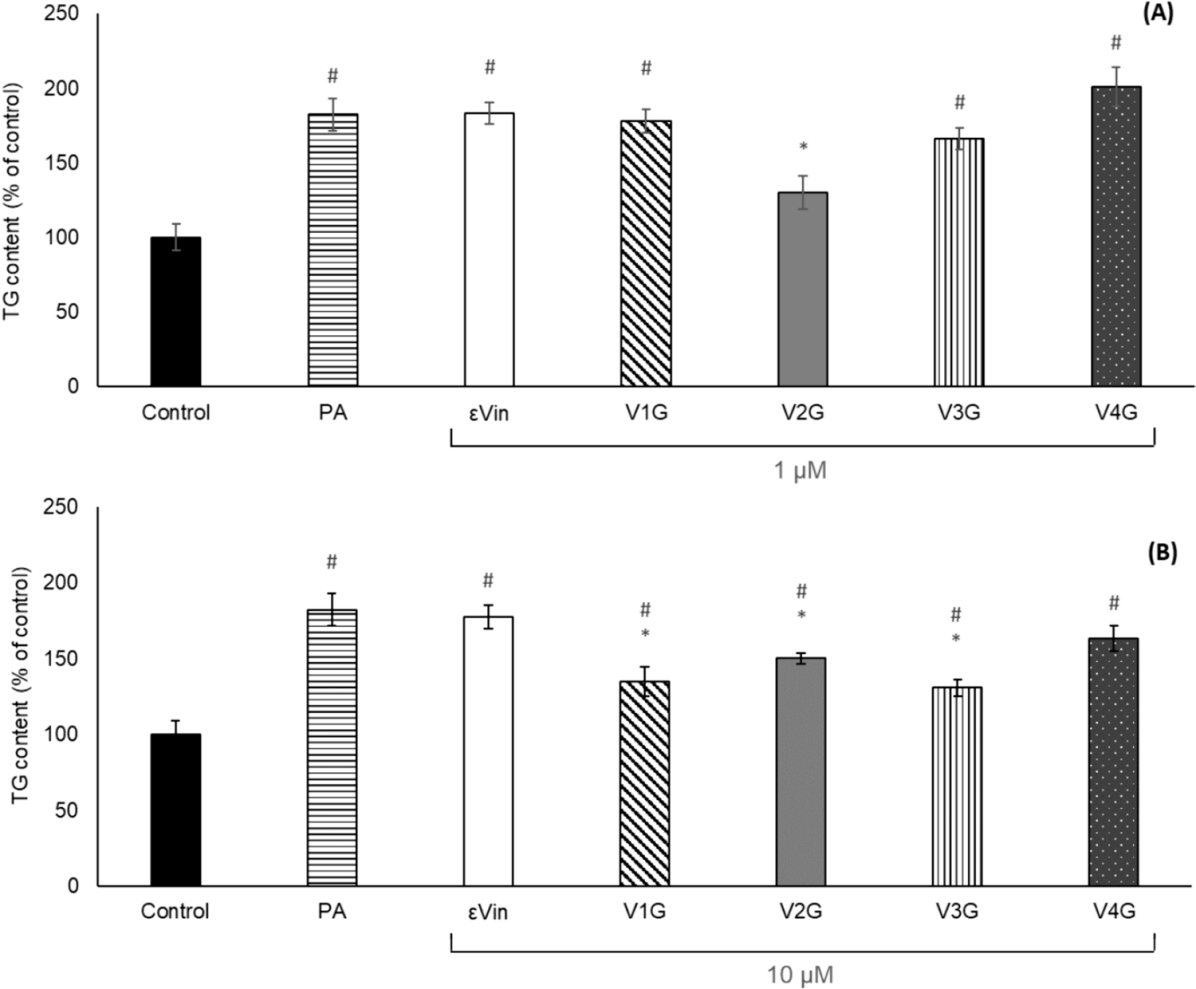
Triglyceride accumulation in AML-12 hepatocytes exposed to palmitic acid (PA) with or without *trans*-ε-viniferin (ε-Vin), *trans*-ε-viniferin-1-glucuronide (V1G), *trans*-ε-viniferin-2-glucuronide (V2G), *trans*-ε-viniferin-3-glucuronide (V3G), or *trans*-ε-viniferin-4-glucuronide (V4G) at 1 µM (A) or 10 µM (B) for 18 h. Data are expressed as mean (% of control) ± SEM. #: significantly different *versus* the control cells (^#^*P* < 0.05), *: significantly different *versus* PA (*P < 0.05). TG: triglyceride

Figure 3 represents the triglyceride content in AML-12 hepatocytes after their incubation with PA, and each molecule at 1 or 10 µM for 18 h. The incubation of cells with 300 µM of PA induced a significant increase in triglyceride accumulation in comparison with the control group (82%), thus verifying the in vitro model of hepatic steatosis. At 1 µM, the parent compound (*trans*-ε-viniferin) had no significant effect on triglyceride content of cells. In the case of the glucuronide metabolites, V2G was able to significantly decrease triglyceride accumulation by 52%, comparing to triglyceride content in PA-treated cells. At 10 µM, *trans*-ε-viniferin remained ineffective but three of the four metabolites (V1G, V2G and V3G) showed significant anti-steatotic effects due to their decreasing triglyceride content by 47%, 32% and 51%, respectively.

### Effects of V2G on the expression of genes involved in triglyceride metabolism

**Fig. 4:**
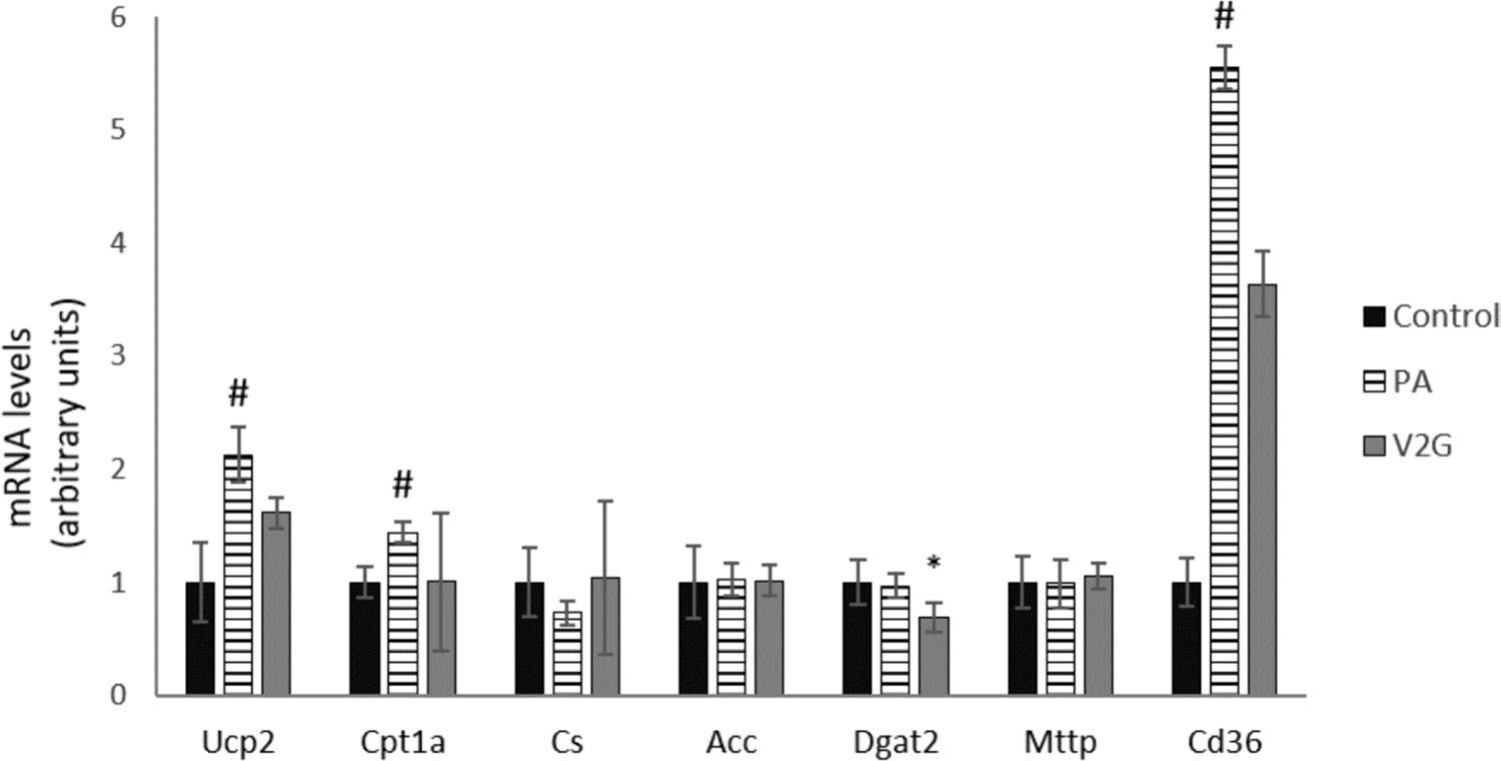
mRNA levels of *Ucp2*, *Cpt1a*, *Cs*, *Acc*, *Dgat2*, *Mttp* and *Cd36* genes (expressed as arbitrary units) in AML-12 hepatocytes exposed to palmitic acid (PA) with or without *trans*-ε-viniferin-2-glucuronide (V2G) at 1 µM for 18 h. Data are expressed as mean ± SEM. #: significantly different *versus* the control cells (*P* < 0.05), *: significantly different *versus* PA-treated cells (*P* < 0.05). Ucp2: uncoupling protein 2; Cpt1a: carnitine palmitoyl transferase 1a; Cs: citrate synthase; Acc: acetyl-CoA carboxylase; Dgat2: diacylglycerol acyltransferase 2; Mttp: Microsomal Triglyceride Transfer Protein; Cd36: cluster of differentiation 36

The gene expression study was carried out in an attempt to determine the mechanisms of action of V2G, the glucuronide metabolite of *trans*-ε-viniferin, which appeared to be the most active in reducing triglyceride accumulation at 1 µM. The expression of seven genes involved in lipid metabolism was measured: *Ucp2, Ctp1a, Cs, Acc, Dgat2, Mttp* and *Cd36* (Fig. 4). The treatment of cells with 300 µM of PA resulted in a significant increase in the expression of *Ucp2, Ctp1a* and *Cd36*, although no significant changes were observed in *Cs, Acc, Dgat2* and *Mttp*. On the other hand, cells incubated with PA and V2G at 1 µM decreased the expression of *Dgat2* and tended to reduce the expression of *Cd36* in comparison to PA-treated cells (35% of reduction; *P* = 0.1).

### Effects of V2G on the expression of proteins involved in fatty acid transport and lipogenesis in AML-12 hepatocytes

**Fig. 5:**
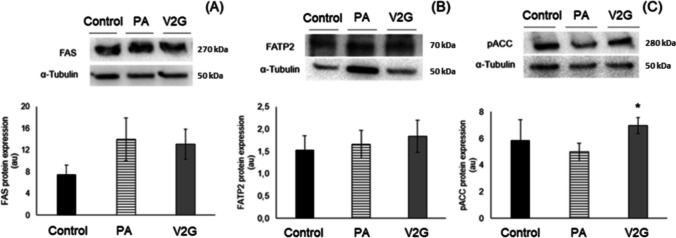
Protein expression of FAS (**A**), FATP2 (**B**) and pACC (**C**) in AML-12 hepatocytes exposed to palmitic acid (PA) with or without *trans*-ε-viniferin-2-glucuronide (V2G) at 1 µM for 18 h. The Western Blot bands shown are representative of six samples per group. Data is expressed as mean ± SEM. *: significantly different *versus* PA (*P* < 0.05). FAS: fatty acid synthase; FATP2: fatty acid transporter protein 2; pACC: phosphorylated acetyl-CoA carboxylase

The incubation of hepatocytes with PA did not have statistically significant effects on the expression of FAS, FATP2 and Ser79 pACC (Fig. 5). The expression of pACC was increased after cell treatment with V2G (Fig. 5C).

### Effect of V2G on FAS enzymatic activity

**Fig. 6:**
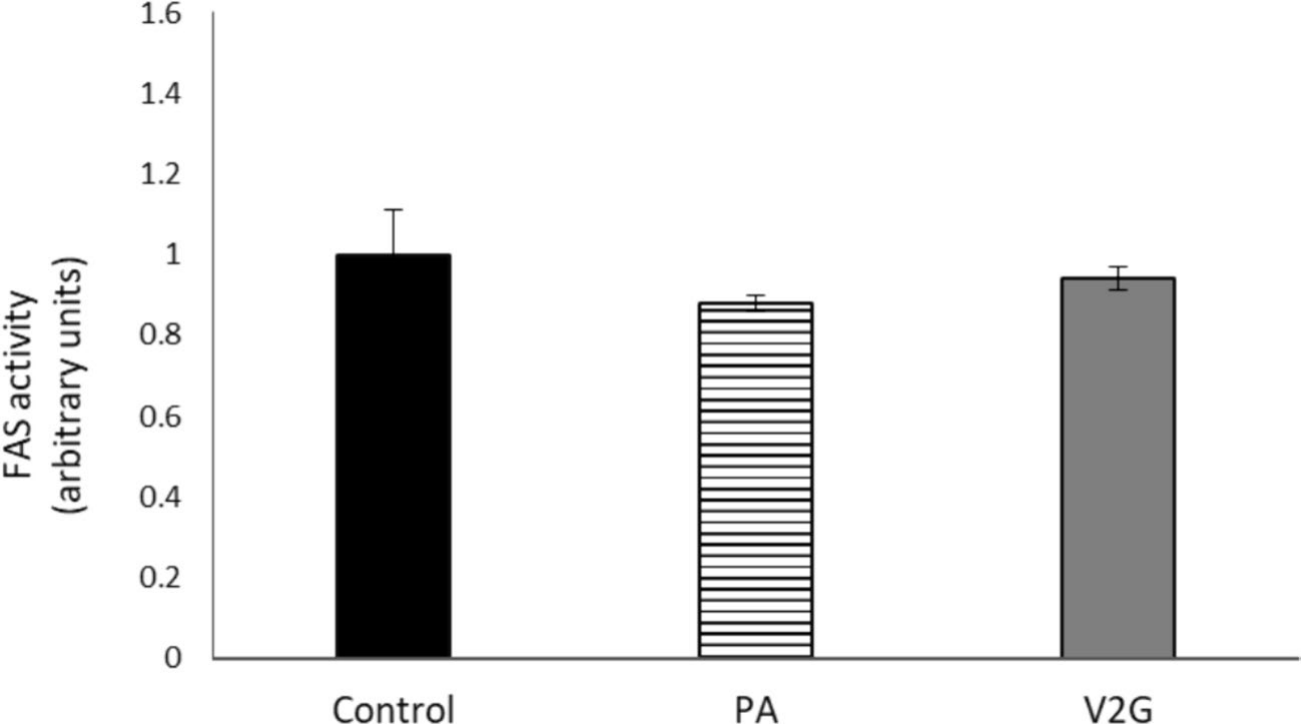
FAS activity (expressed as arbitrary units) in AML-12 hepatocytes exposed to palmitic acid (PA) with or without *trans*-ε-viniferin-2-glucuronide (V2G) at 1 µM for 18 h. Data is expressed as mean ± SEM

Although protein expression of FAS was not modified by V2G, its activity was determined in order to dismiss a potential post-translational regulation mechanism. We observed that V2G did not change the activity of this enzyme (Fig. 6).

### Metabolic profile *of media* and hepatocytes after cell treatment

**Fig. 7:**
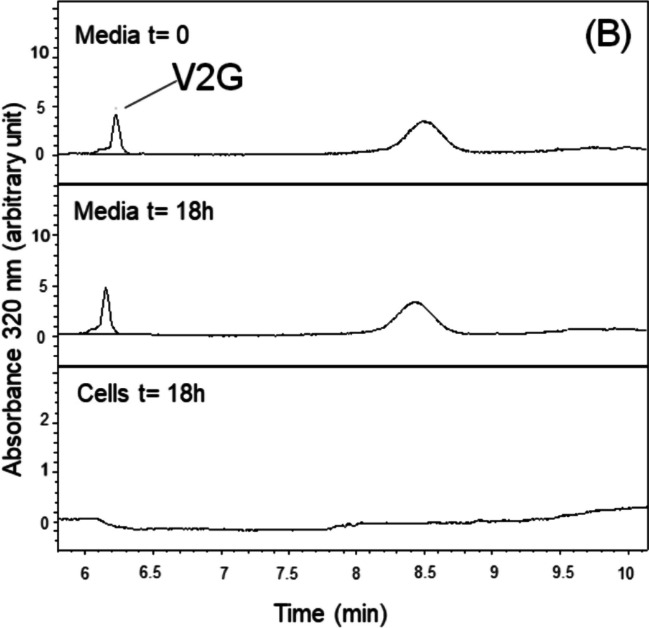
Chromatogram UPLC-DAD at 320 nm of media at t = 0 and media and cells at t = 18 h after the incubation of AML-12 hepatocytes exposed to palmitic acid (PA) with or without *trans*-ε-viniferin (ε-Vin) or *trans*-ε-viniferin-2-glucuronide (V2G) at 10 µM

The cell incubation media as well as the hepatocytes were analysed in UPLC-DAD at 320 nm. For each compound (at 10 μM), the chromatograms of cell media before (t = 0) and after (t = 18 h) incubation and the chromatograms of cells after incubation (t = 18 h) are shown in Fig. 7. After 18 h of AML-12 hepatocyte incubation with *trans*-ε-viniferin, no *trans-*ε-viniferin glucuronides were detected in the incubation media. Furthermore, *trans-*ε-viniferin was found in the cell samples, indicating that *trans-*ε-viniferin can either enter the cell or be on their surface. When cells were incubated with V2G (selected among all the glucuronide metabolites because it is the main metabolite in vivo and the most effective in the present study)*,* the concentration of V2G in the incubation media did not vary after 18 h, and no other compounds appeared on the chromatogram. Moreover, no V2G was detected in cells. Nevertheless, the presence of small amounts below the detection limit of the chromatographic technique cannot be ruled out.

### Database analysis of differences in UGTs isoform expression between AML-12 and primary murine hepatocytes

**Fig. 8:**
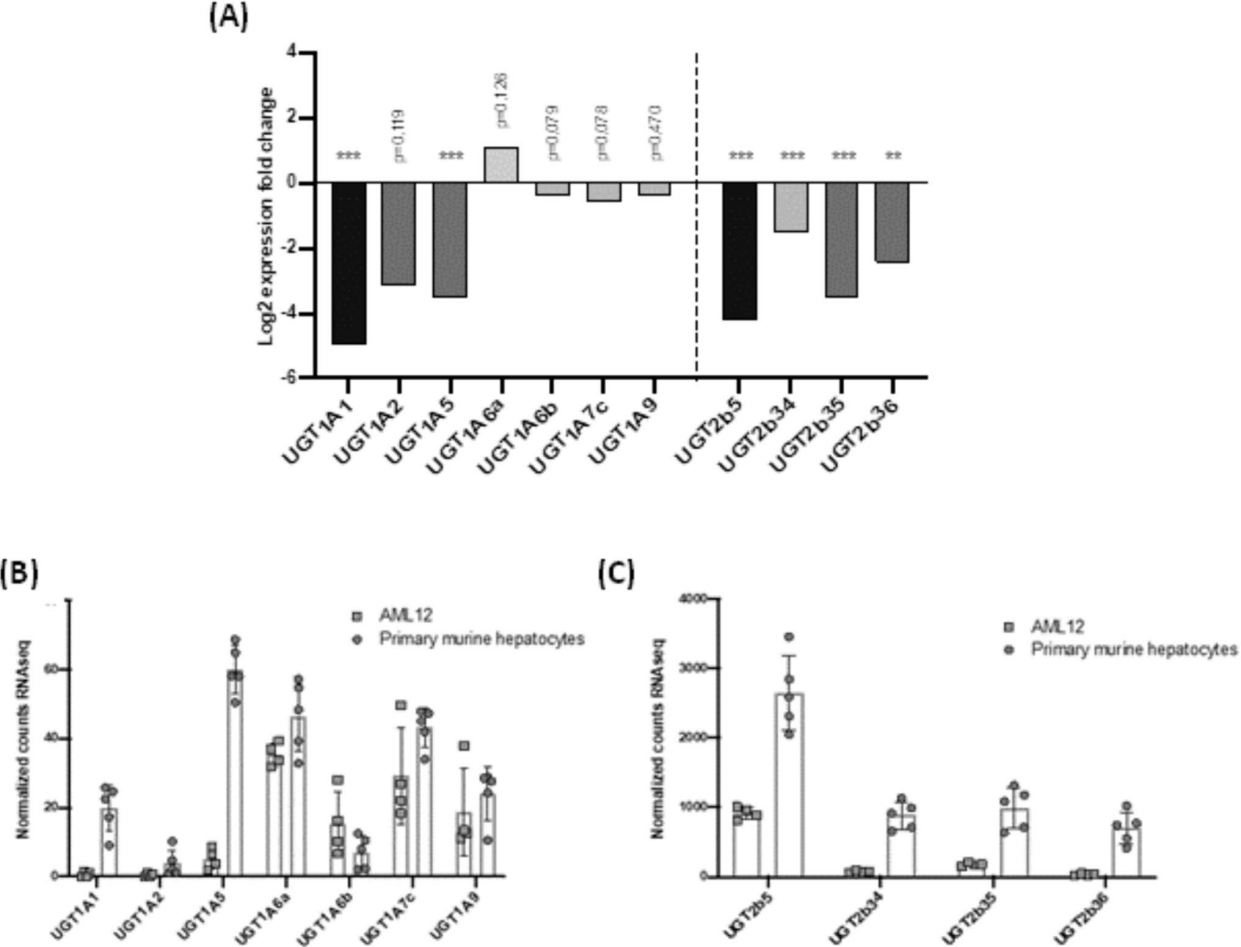
Differential expression of UGT1A and UGT2b genes in AML-12 cells and primary murine hepatocytes**.** Log2 expression fold changes of *UGT1A and UGT2b genes* between AML-12 cells and primary murine hepatocytes in GEO GSE171184 dataset (**A**). RNA-seq normalised counts for *UGT1A* (**B**) and *UGT2b* (**C**) in AML-12 cells and primary murine hepatocytes in GEO GSE171184 dataset. ** *P* < 0.01; *** *P* < 0.001

In order to compare *UGT* isoform expression profile in AML-12 cells with that of murine primary hepatocytes, we used online GEO database and identified a study that performed transcriptomic analyses on these two cell types. According to the RNA-seq data analysis, UGT expression profiles differ between the two cell types. In particular, *UGT1A*, *UGT1A5*, *UGT2b5*, *UGT2b34*, *UGT2b35* and *UGT2b36* gene isoforms were significantly less expressed in the immortal hepatocyte AML-12 cell line than in the primary hepatocytes. In contrast, no significant difference was found for *UGT1A2*, *UGT1A6a*, *UGT1A6b*, *UGT1A7c* and *UGT1A9* genes (Figs. 8; A, B and C).

## Discussion

*Trans-*ε-viniferin is a stilbene found in relatively large amounts in vine roots, sometimes in higher concentrations than resveratrol [6]. In vivo studies in rodents have shown protective properties against hepatic steatosis for this compound [30]. However, *trans-*ε-viniferin is poorly bioavailable [25], especially due to its high glucuronidation, a phase II metabolism biotransformation [11, 12]. For this reason, we hypothesised that the described beneficial effects attributed to *trans-*ε-viniferin could be exerted specifically by its metabolites. Consequently, in order to better understand the ε-viniferin anti-steatotic effect previously observed in rats, the aim of the present study was to determine the potential anti-steatotic activity of the main phase II metabolites of *trans*-ε-viniferin in cultured murine hepatocytes. As the in vivo anti-steatotic effect was observed in a rodent model, for the present in vitro study, the murine AML-12 cell line was chosen. Nevertheless, to analyse their effect in a human cell line such as HepG2 could be interesting in a future approach.

To our knowledge, this is the first time that the bioactivity of *trans*-ε-viniferin metabolites is addressed. At this point, it is important to highlight that the study of the effect of *trans*-ε-viniferin metabolites in vivo is not currently possible, as the metabolites are not commercially available. On the other hand, the amount of metabolites that we are able to produce by hemisynthesis is not high enough to address an in vivo study.

At 1 µM, whereas *trans*-ɛ-viniferin did not show an anti-steatotic effect, the metabolite V2G was active, and prevented completely the effects of PA exposure insofar as cell triglyceride content was not different from the control condition without PA incubation. At 10 µM, *trans-*ε-viniferin remained ineffective, although three of the four *trans-*ε-viniferin glucuronide metabolites were active. The fact that, in the case of V2G, the effect induced by the lowest dose (1 μM) was higher than that induced by a higher dose (10 μM) had been previously observed in adipocytes treated with quercetin and its metabolites [14, 15]. Furthermore, in another study kaempferol significantly reduced lipid accumulation in maturing pre-adipocytes treated at 1, 10 and 25 µM, but this effect was more prominent in cells treated at the two lowest doses [20]. This phenomenon has also been observed in vivo, in a study carried out in mice fed with an obesogenic diet and treated with resveratrol, meaning that this is a quite common response pattern to polyphenols, or at least to some of them [9]. Interestingly, V2G and V3G, two of the four metabolites analysed, are those found in the plasma of rats administered with *trans*-ε-viniferin [11]. Moreover, after incubation of liver fractions with ε-viniferin, V2G and V3G were the main glucuronide metabolites [12]. In addition, we have recently shown that after oral administration of 20 mg/kg body weight of *trans*-ε-viniferin to rats, the plasma Cmax for glucuronides is 1.7 ± 1.1 µM, a value close to 1 µM tested in the present study [5].

It has been shown that *trans*-ε-viniferin shows biological properties after oral administration. On the other hand, the concentration of glucuronide metabolites was up to 108 times higher in the blood and more than 60 times higher in the liver after oral administration of the molecule [5]. In addition, in the present study, whereas *trans*-ε-viniferin was ineffective, its metabolites were able to reduce the triglyceride amount in hepatocyte cultures. Taking all that into account, it seems that, in fact, the main responsible for the effects of *trans*-ε-viniferin in the liver can be the glucuronide metabolites.

The analysis of the contents of culture media and cells after incubation with *trans*-ε-viniferin provided us with information on the metabolism of this dimer in AML-12 cells. Indeed, no metabolism of the stilbene was observed in these cells because no glucuronides were found neither in the incubation media nor in the hepatocytes when these cells were incubated with *trans*-ε-viniferin for 18 h. According to previous results obtained in vitro in the presence of liver protein extracts, and in in vivo studies, where this molecule was largely glucuronidated, the glucuronidation of *trans*-ε-viniferin after cell incubation for 18 h should be expected, but this reaction did not occur. These results are even more surprising considering that AML-12 cells are able to glucuronide resveratrol, of which *trans-*ε-viniferin is a dimer [4]. A possible explanation could be that AML-12 cells do not have the UGT isoforms that metabolise *trans*-ε-viniferin. Indeed, it has been shown that different isoforms of UGTs glucuronide the two isomers of resveratrol, UGT1A6 for *cis*-resveratrol and UGT1A1 for the *trans*-isomer. In this scenario, we decided to analyse whether the expression of the enzymes responsible for *trans*-ε-viniferin metabolization could be differently expressed in primary murine hepatocytes and the AML-12 cell line, using public available RNA-seq data. The comparison showed that the cultured cells expressed significantly less *UGT* gene isoform. Therefore, we hypothesised that this cell line could not metabolise *trans*-ε-viniferin due to the different *trans*-ε-viniferin glucuronidation pattern between AML-12 hepatocytes and murine primary hepatocytes, which could explain the different results obtained in comparison to in vivo studies. Interestingly, this fact makes AML-12 hepatocytes a valuable tool for the present study. The fact that *trans*-ε-viniferin was not metabolised and that it showed no effect on triglyceride accumulation certainly allows us to speculate that the glucuronides of *trans*-ε-viniferin are probably the active molecules in vivo.

Concerning V2G, we observed a pronounced triglyceride reduction after the treatment of cells, but we did not find a quantifiable amount of this compound inside cells. Although this fact was also surprising, we hypothesised that the effect of the molecule could be given via interaction with cell plasma membrane. Indeed, binding sites for resveratrol and other phenolic compounds in cell plasma membrane have been reported. Bastianetto et al. (2009) identified binding sites for resveratrol in the plasma membrane fraction of rat brain; in fact, binding sites for resveratrol in the membrane were more abundant than in nucleus or other organelles [3]. In another study aimed to analyse the molecular mechanisms of epigallocatechin-gallate, the authors observed that the trans-membrane receptor 67LR showed a high affinity to this molecule [37]. In addition, it has been stated that interactions of flavonoids with the lipid bilayer are responsible for some of their biological activity, such as antioxidant or anticancer effects [24, 34, 35].

In view of the activity of V2G, the most abundant metabolite in vivo, we decided to carry out gene and protein expression, and enzyme activity analyses in order to define the mechanism of action underlying the preventive effect of this molecule in hepatocytes. After gene expression analysis, we observed a decrease in mRNA levels of *Dgat2*, meaning that a decline in triglyceride assembly could be involved in the observed reduction in triglyceride accumulation. Moreover, although only a tendency in the reduction of *Cd36* gene expression was observed, the percentage of reduction was of 35%. It has been stated that CD36 is directly involved in the development of fatty liver in an elevated fatty acid situation due to its role in fatty acid entry to hepatocytes [16]. Taking all this into account, a reduced uptake of PA from the media may have been involved in the anti-steatotic effects of V2G observed in the present study. On the other hand, a significant increase in pACC protein expression was found in the cells incubated with this metabolite. As the phosphorylated form of ACC, the limiting enzyme in de novo lipogenesis, is the inactive form of the enzyme, this result shows that this enzyme was inhibited by V2G, and thus, that a decrease in fatty acid synthesis from acetyl-CoA could be partially mediating the effect of this metabolite. In a study carried out by Hung et al*.* (2019), 3T3-L1 adipocytes exposed to *trans*-ε-viniferin increased the phosphorylation of AMPK [22], which, once phosphorylated, catalysed the phosphorylation of ACC. This study gives support to the hypothesis that V2G can act by inhibiting de novo lipogenesis.

In addition to the anti-steatotic effect of ɛ-viniferin, seemingly mediated by its metabolite V2G, in vitro studies have shown anti-inflammatory and antioxidant properties of this resveratrol dimer [30]. Taking into account that oxidative stress and inflammation are key alterations in liver steatosis and its progression to liver steatohepatitis, all these effects collectively support the proposal of *trans*-ε-viniferin as a useful phenolic compound to prevent and/or to treat MAFLD. Nevertheless, it is important to bear in mind that this experiment has been carried out in murine cells, so future experiments are needed in order to confirm that these promising can be extrapolated to human hepatocytes.

## Conclusion

In conclusion, some of the tested glucuronide metabolites of *trans*-ε-viniferin prevent triglyceride accumulation in hepatocytes, V2G being the most efficient. This effect is mediated, at least in part, by the inhibition of de novo lipogenesis, fatty acid uptake and triglyceride assembly. This data, together with the lack of effect of the parent compound, suggest that the anti-steatotic activity of *trans*-ɛ-viniferin observed in rats is mediated by its metabolites. Therefore, the low bioavailability of the parent compound does not necessarily represent a problem when it comes to using *trans*-ε-viniferin in the elaboration of nutraceuticals or functional food.

## Data Availability

Data sharing is not applicable to this article as no new data were created or analyzed in this study.
